# Evaluation of Head Posture in Wheelchair‐Bound Individuals and Its Relation to Malocclusion: A Proof‐of‐Concept Study

**DOI:** 10.1111/ocr.70098

**Published:** 2026-01-14

**Authors:** Kelly Billiaert, Mustafa Al‐Yassary, Stavros Kiliaridis, Gregory S. Antonarakis

**Affiliations:** ^1^ Division of Orthodontics, University Clinics of Dental Medicine University of Geneva Geneva Switzerland; ^2^ Department of Orthodontics University of Bern Bern Switzerland

**Keywords:** head posture, malocclusion, wheelchair

## Abstract

**Background:**

Malocclusions are significantly more prevalent among individuals with physical and mental disabilities. Head posture, particularly in wheelchair‐bound individuals, may play a role in craniofacial growth and occlusal development, but its specific impact remains poorly understood.

**Objective:**

To evaluate head posture in wheelchair‐bound individuals and assess potential correlations with malocclusion characteristics.

**Methods:**

Twenty wheelchair‐bound participants (aged 6–30 years) were categorised into three groups based on their head control (full, partial, or no control). Head posture was dynamically measured on three axes (pitch, roll and yaw) using an Inertial Measurement Unit for precise 3D tracking. Malocclusions, including molar and canine sagittal relationships, overjet, overbite and crossbites, were assessed through standardised clinical examinations.

**Results:**

All wheelchair‐bound participants showed significantly greater deviations in head posture compared to controls. The roll axis differed significantly across groups (*p* = 0.004), with the no control group exhibiting the greatest deviation. A significant correlation was observed between pitch axis deviation and sagittal molar relationships (*r* = −0.59, *p* = 0.006), indicating that a backward head posture was associated with Class II malocclusion, while a forward head posture was linked to Class III malocclusion. No significant correlations were found for the roll or yaw axes.

**Conclusion:**

Significant head posture deviations were detected in wheelchair‐bound individuals, with those having no head control showing more extreme deviations. This study found significant correlations between antero‐posterior head posture deviations and sagittal molar relationships in wheelchair‐bound individuals, suggesting that head posture could be a contributing factor to malocclusions in this population. Given the limited sample, these results should be interpreted cautiously.

## 1 Introduction

Individuals with physical or mental disabilities exhibit a significantly higher prevalence of malocclusions [1]. Previous studies have reported that less than half of individuals with disabilities present normal occlusion or minor malocclusions that do not require treatment [2]. Among this population, open bite, posterior crossbite and anterior crossbite are the most common types of malocclusions [3]. Individuals with intellectual disabilities are particularly at risk of developing malocclusions, partly due to altered oral functions such as mastication, swallowing and tongue posture [4, 5].

The development of malocclusions is multifactorial, involving genetic, neuromuscular and environmental influences including compromised oral functions. Mouth breathing related to airway obstruction can alter tongue position and orofacial muscle function [6, 7], while parafunctional habits such as bruxism and thumb sucking, more frequent in this population, further contribute to the development of malocclusions [8, 9]. Additionally, genetic conditions associated with disabilities frequently involve craniofacial anomalies and malocclusion [10].

Head posture has been related to the development of malocclusions; research on craniofacial development based on lateral cephalometric radiographs suggests that head positioning can affect vertical growth patterns. Individuals who maintain a hyperextended head posture, such as in patients with obstructive sleep apnoea, or those who are blind, often exhibit a long facial structure, marked by greater anterior facial height growth and diminished posterior facial height growth [11, 12]. Despite these findings, few studies have examined head posture in individuals who rely on wheelchairs for daily mobility, a group in which head positioning is often constrained by seating configurations and neuromuscular control.

The elevated prevalence of malocclusion and limited access to orthodontic care among individuals with disabilities [4] underscore a substantial need for orthodontic treatment, including appropriate preventive measures in order to prevent or at least reduce the severity of the maloclussions witnessed. When considering weelchaair‐bound individuals, anecdotal observations bring to light the potential problem of head posture, especially with inadequately adapted headrests. Wheelchairs and headrests much be customised for each patient, according to their unique needs. Improper head positioning may not only be related to the headrest itself but can also stem from the body's alignment in the wheelchair. Inadequate head support may lead to compensatory postures that disrupt oral functions such as mastication, swallowing, breathing and speech potentially influencing occlusal development. Proper customization of wheelchairs and headrests is therefore essential, as misalignment can exacerbate both postural and orofacial dysfunctions.

Given the multifactorial aetiology of malocclusions in this population, the present proof‐of‐concept study focuses on head posture as a potential contributing factor. The aim of this cross‐sectional study was to evaluate three‐dimensional head posture in wheelchair‐bound individuals with varying levels of head control and to assess its possible association with malocclusion characteristics. As a proof‐of‐concept study, by comparing individuals with varying degrees of head control, the present investigation sought to determine whether impaired head posture may be a contributing factor in the development of malocclusions.

## 2 Material and Method

### 2.1 Study Design

The present cross‐sectional study was approved by the local research ethics committee (CCER 2019‐00659) and conducted in accordance with the Declaration of Helsinki. The study was designed and reported with the STROBE guidelines [13] in mind to ensure methodological rigour, transparency and completeness in reporting. Given the exploratory nature of the present study, it was conceived as a proof‐of‐concept investigation aimed at generating preliminary evidence.

### 2.2 Setting

This study was conducted in various institutions for individuals with disabilities across the French‐speaking region of Switzerland (Swiss Romandy). Data were collected during scheduled visits to these institutions over a three‐year period (from 2020 to 2023). The participating institutions included both residential facilities (specialised care homes) and day‐care structures (specialised day facilities), representing a heterogeneous population with diverse medical, neuromuscular and syndromic conditions requiring wheelchair use. All visits were carried out following a standardised protocol to ensure consistency in data collection across all sites.

### 2.3 Participants

Individuals were eligible for inclusion in the study if they were aged 6 years or older, in the early mixed, late mixed, or permanent dentition and had grown up using a wheelchair as their primary means of mobility. All eligible individuals present in the participating institutions during the study period were invited to take part. Informed consent was obtained from participants or their legal guardians, depending on age and cognitive ability.

Participants were categorised into three distinct groups based on their degree of head posture control. The first group comprised wheelchair‐bound individuals demonstrating full control of their head posture, not requiring the support of a headrest (full head control group). The second group comprised wheelchair‐bound individuals with partially limited head control who had headrests and used them when needed (partial head control group), while the third group was made up of wheelchair‐bound individuals with no voluntary head posture control, fully dependent on a headrest for head positioning (no head control group).

A previously collected control dataset consisting of 20 healthy non‐wheelchair‐bound young adults was used for comparison [14]. These control measurements were obtained with the same IMU system, calibration protocol and testing environment, ensuring methodological equivalence.

The inclusion of this control group allowed deviations in head posture to be expressed relative to established normative values, improving interpretability.

The division of the sample into three distinct groups aimed to assess whether head position control achieved spontaneously or provided by the headrest (partially or completely) influences dentoalveolar growth and potentially contributes to the development of malocclusions.

Only a modest sample size was aimed for, reflecting the proof‐of‐concept nature of the study and the limited eligible population available in the participating institutions. Although no power calculation was performed due to this study approach, this limitation is acknowledged and the results interpreted cautiously.

The data collected from the wheelchair‐bound individuals (and from their parents or caretakers) were derived from a questionnaire, a clinical examination and an objective head posture assessment.

### 2.4 Questionnaire

As part of the visits to the various institutions, a questionnaire was developed to gather key information about the participants. The questionnaire focuses on the individual's medical history, including the reasons for their use of a wheelchair.

Additionally, the questionnaire included questions regarding the participant's habitual posture in the wheelchair, including the position of their head. Lastly, questions concerning their dietary habits and oral hygiene practices were incorporated.

### 2.5 Clinical Examination

A clinical intraoral examination was performed using an intraoral mirror and a periodontal probe, with appropriate lighting, allowing for a comprehensive assessment of intermaxillary occlusal relationships and the presence of malocclusions. Clinical assessment included sagittal molar relationships (Angle classification), overjet, overbite, and the presence any anterior and/or posterior crossbites. Additionally, an assessment of the general dental condition, including the detection of carious lesions and signs of periodontal disease was carried out.

### 2.6 Head Position Assessment

Inertial measurement units (IMUs) were utilised to measure head position in three dimensions (pitch, roll and yaw). IMUs are electronic devices consisting of components such as a gyroscope, an accelerometer and a sensor that measures angular rate and gravity, that enable precise tracking of movements over a certain period of time and were chosen for their ease of use and accuracy [14, 15, 16]. With its use, a dynamic recording of head position over extended periods of time is achieved, making it possible to analyse the full range of natural head posture (NHP) variations during the recording.

The IMU system that was used for measuring head position was the Xsens Movella DOT Motion Tracking system (Movella Technologies B.V., Enschede, Netherlands), a wearable sensor developed for human kimenatic analysis. Before starting the measurements, the IMU was calibrated to define the neutral position (set to 0° on all axes). This process involved placing the sensor on the ground in front of the participant, ensuring it was perpendicular to the ground. After appropriate calibration, the sensor was moved to the participant's forehead for the actual dynamic head posture measurements. Reproducibility was enhanced as best as possible by controlling sensor placement and instructions given to the individuals, properly performing calibration procedures before use on each individual, and previous evaluation of the reliability and validity of the system [15, 16].

### 2.7 Statistical Analysis

Following confirmation of normal distribution of the data (using Shapiro–Wilk tests), parametric statistical analyses were used. The parameters of malocclusion were evaluated according to their nature. Continuous variables were used for the measurement of overjet and overbite recorded in millimetres. Binary variables were used to assess the presence or absence of specific malocclusions such as anterior and posterior crossbite (coded 1 for presence and 0 for absence). The sagittal molar relationships were transformed into a continuous variable for analytical purposes: 1 for Class II, 2 for Class I and 3 for Class III malocclusion.

Head posture deviations (in the three axes separately; namely pitch, roll and yaw) for the three experimental wheelchair‐bound groups were assessed in respect to the number of standard deviations from the norm (the norm and standard deviation being defined by the healthy control group). These head posture measurements were therefore expressed relative to reference population norms as deviations in standard deviations, consistent with what is sometimes carried out in research on rare diseases [17, 18, 19]. A one‐way ANOVA, with post hoc tests (Tukey and Bonferroni), was conducted to examine whether the head posture axis (pitch, roll and yaw) varied significantly across the different experimental wheelchair‐bound groups.

Paired sample *t*‐tests were conducted within each wheelchair‐bound group to assess whether statistically significant differences existed between the different axes (pitch, roll, yaw) to assess which axis showed more variation in each group. Paired correlations were also assessed to determine whether deviations in one axis were associated with simultaneous deviations in any of the other measured axes.

To explore the relationships between head posture axes (pitch, roll, yaw) and intermaxillary occlusal characteristics (sagittal molar relationships, overjet, overbite and the presence or absence of crossbite), Pearson correlations were carried out on the whole wheelchair‐bound experimental sample. All statistical analyses were performed using SPSS (IBM SPSS Statistics for Windows, version 29, Armonk, NY, USA) and a threshold of *p* < 0.05 was considered as statistically significant.

## 3 Results

### 3.1 Participants

The experimental sample of the study consisted of 20 children, adolescents and young adults aged 6 years and older, 6 of whom were classified into the full control group, 7 in the partial control group and 7 in the no control group (Table 1). The control group was composed of 20 young adults (Table 1).

**TABLE 1:** Baseline characteristics of the study population.

Groups	Total	Females	Males	Age range (years)	Mean age (years)
Full head control	6	3	3	9–19	12.5
Partial head control	7	4	3	6–29	15.7
No head control	7	4	3	15–33	19.7
Control group	20	13	7	20–30	23.6

### 3.2 Head Posture in Different Groups of Participants

The control group exhibited slight average deviations in head posture from the neutral head position (defined as 0° in pitch, roll and yaw measurements), namely −0.2° ± 2.1° for the pitch, 0.4° ± 1.4° for the roll and 0° ± 2.3° for the yaw axes (Table 2). Mean head posture deviations for the three groups of wheelchair‐bound children with varying degrees of head control are also shown in Table 2, where one can notice very large standard deviations.

**TABLE 2:** Mean and standard deviations for head posture in all groups in the three axes.

Group	Pitch (mean ± SD; degrees)	Roll (mean ± SD; degrees)	Yaw (mean ± SD; degrees)
Control group	−0.2 ± 2.1	0.4 ± 1.4	0 ± 2.3
Full head control	5.5 ± 15.8	6.5 ± 7.0	5.7 ± 15.2
Partial head control	−17.9 ± 36.5	−20.9 ± 22.7	1.4 ± 34.4
No head control	−10.1 ± 16.2	−27.4 ± 13.6	−11.9 ± 18.1

**FIGURE 1:**
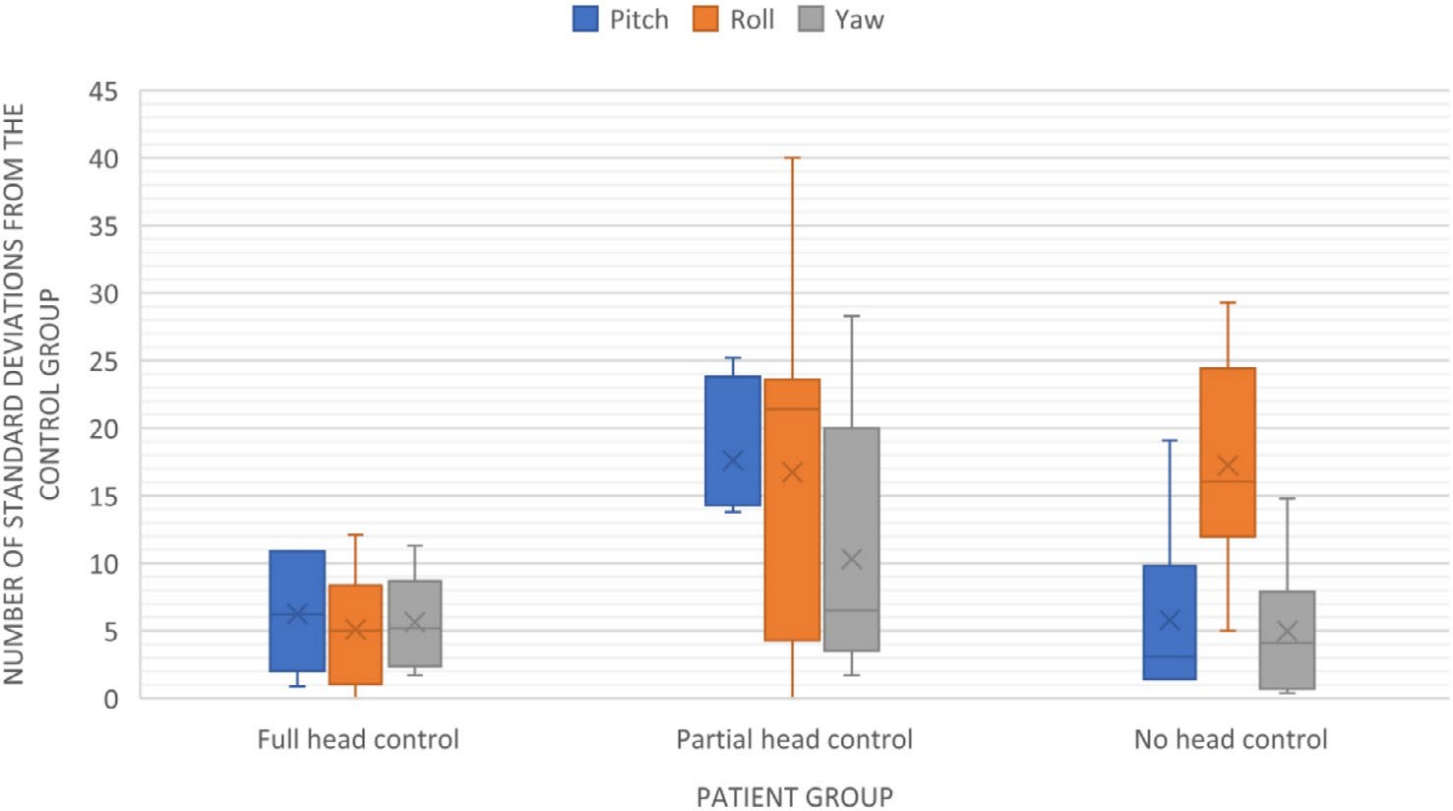
Deviation in head posture (expressed as standard deviation units) for the three groups of wheelchair‐bound individuals.

When looking at head posture deviations in the three experimental wheelchair‐bound groups in respect to the number of standard deviations away from the control group, all wheelchair‐bound individuals exhibited higher deviations in head posture from the neutral head position (Table 3; Figure 1).

**TABLE 3:** Number of standard deviations (SD) from the control group, in the wheelchair‐bound individuals, for head posture in the three axes, expressed as SD units.

Group	Pitch (mean ± SD)	Roll (mean ± SD)	Yaw (mean ± SD)
Full head control	6.3 ± 4.2	5.1 ± 4.4	5.4 ± 3.6
Partial head control	17.6 ± 4.9	16.7 ± 13.9	10.3 ± 10.0
No head control	6.3 ± 6.3	19.6 ± 9.7	6.6 ± 6.5

With regard to differences between the control group and the wheelchair‐bound groups, participants with full head control exhibited the lowest number of standard deviations from the control group, namely 6.3 for pitch, 5.1 for roll, and 5.6 for yaw. Those with partial head control showed an increased number of standard deviations from the control group, for the pitch (17.6), roll (16.7) and yaw (10.3) axes. The no head control group demonstrated the greatest number of standard deviations from the control group for the roll axis (19.6).

Comparisons between different head control groups in the wheelchair‐bound experimental sample showed that the pitch axis was significantly different between the 3 groups (*p* = 0.001), whereby participants with partial head control showed significantly more deviation in the pitch axis compared to the other groups (Table 3; Figure 1). The mean difference in the pitch axis between the full head control group and partial control group was −11.4° (*p* = 0.003) and between the partial control group and the no control group was 11.3° (*p* = 0.003), meaning that participants with partial head control showed significantly more deviation of head posture compared to the full control and no control groups (Table 3; Figure 1). The roll and yaw axes did not show any statistically significant differences between the different wheelchair‐bound groups.

When comparing the 3 axes within each wheelchair‐bound group, for the full head control group, no statistically significant differences or correlations were found between the pitch, roll and yaw axes. In the partial head control group, a significant paired correlation was found between the pitch and roll axes (*p* = 0.013), with deviations in both axes tending to occur simultaneously. In the no head control group, the roll axis was the axis that consistently and significantly showed the greatest amount of deviation when compared to the pitch (*p* = 0.013) and yaw (*p* = 0.015) axes (Figure 1).

### 3.3 Correlation Between Head Posture and Malocclusions

The only deviation in head posture significantly associated with intermaxillary occlusal relationships was deviations in the pitch axis, with good correlation found between pitch deviations and sagittal molar relationships (*r* = −0.59; *p* = 0.006). A backward head posture was correlated with the presence of a Class II malocclusion (with Class II molar relationships), while a forward head posture was correlated with the presence of a Class III malocclusion (with Class III molar relationships) (Table 4).

**TABLE 4:** Correlations between head posture deviations and intermaxillary occlusal relationships in wheelchair‐bound individuals.

Head posture axis	Molar relationships	Overjet	Overbite	Crossbite
Pitch				
Pearson correlation	0.59	0.30	0.14	0.39
Significance (*p* value)	0.006	0.19	0.56	0.08
Roll				
Pearson correlation	0.26	0.36	−0.17	0.018
Significance (*p* value)	0.27	0.12	0.94	0.94
Yaw				
Pearson correlation	0.01	0.26	−0.25	0.06
Significance (*p* value)	0.96	0.27	0.28	0.79

## 4 Discussion

The present study investigated the association between head posture and malocclusions in wheelchair‐bound individuals with varying degrees of head control. Our findings indicate that among the three axes of head posture (pitch, roll and yaw), only the pitch axis showed a statistically significant correlation with intermaxillary molar relationships, meaning that those with a more severe backward head posture tended to have Class II molar relationships while those with more severe forward head posture tended to have Class III molar relationships.

These findings are consistent with previous cephalometric studies [7, 12] suggesting that craniofacial growth patterns may be influenced by head positioning, but the present study suggests this for populations with more extreme head posture deviations and using more sophisticated and three‐dimensional methodology. Prolonged extension or flexion of the head can possibly affect mandibular positioning and subsequently alter occlusal development during critical growth periods. Additionally, neuromuscular compensation mechanisms may shift mandibular postural position in response to sustained head posture imbalances, thereby influencing sagittal dental relationships [9, 20, 21].

While the roll and yaw axes showed observable deviations in wheelchair‐bound individuals with partial or no head control, their correlation with occlusal characteristics was weak and not statistically significant. This implies that the roll and yaw axes may have less of a direct influence on dentoalveolar development compared to the pitch axis. Alternatively, it is possible that their effects on occlusion are secondary to or masked by other factors, such as neuromuscular tone, orofacial function, or compensatory behaviors.

When looking at head posture in wheelchair‐bound individuals with varying degrees of head control, it was revealed that the roll axis differed significantly across groups. Participants with full head control exhibited significantly less lateral head deviation compared to those with partial or no control. While roll itself was not correlated with specific occlusal features, this finding supports the notion that headrest dependency may be associated with substantial postural misalignment.

These results support the notion that altered head posture, particularly in the antero‐posterior plane (pitch axis), may influence sagittal molar relationships in wheelchair‐bound individuals. Recent research in special‐needs populations has highlighted the interplay between postural instability, impaired orofacial muscle coordination and altered mandibular function, suggesting that neuromuscular deficits may amplify the impact of sagittal head deviations on occlusal development [21].

Head posture may thus be one of the contributing factors to the development of malocclusions, especially in individuals with limited head postural control. Clinical assessment of head posture should thus be integrated into orthodontic evaluations for wheelchair‐bound individuals with disabilities. Early identification and correction of postural issues could be a preventive strategy to limit the development or progression of malocclusions in this population. Multidisciplinary approaches to optimise body and head alignment through customised wheelchairs and regular postural assessments may represent a non‐invasive adjunct to orthodontic treatment.

The primary limitations of the present study were the relatively small sample size, the cross‐sectional design, and the heterogeneous aetiologies of the disabilities and ages of the individuals (acting as possible confounders) which limit generalizability and statistical power. As a proof‐of‐concept study, the finding should be interpreted cautiously, and future studies with larger cohorts are needed to confirm these associations.

Longitudinal studies tracking head posture and occlusal development over time would also be needed in order to establish whether early postural interventions could potentially reduce the incidence or severity of malocclusion in this population.

## 5 Conclusions

Important head posture deviations were detected in wheelchair‐bound individuals with different levels of head control compared to a sample of seated healthy individuals, with those with no head control showing more extreme deviations.

Significant correlations were found in the present sample between antero‐posterior head posture deviations and sagittal molar relationships in wheelchair‐bound individuals, whereby a more backward head posture was associated with Class II malocclusions while a more forward posture was associated with Class III malocclusions.

No significant associations were found between the presence of malocclusions and left–right head posture deviations or head tilt deviations.

## Author Contributions

**Kelly Billiaert:** investigation, data curation, formal analysis, writing – original draft. **Mustafa Al‐Yassary:** investigation, data curation, formal analysis, writing – original draft. **Stavros Kiliaridis:** conceptualization, methodology; writing – review and editing. **Gregory Antonarakis:** conceptualization, methodology, writing – review and editing; supervision.

## Funding

The authors have nothing to report.

## Ethics Statement

This study was approved by the local Research Ethics Committee (Commission cantonale d'éthique de la recherche [CCER], approval number: 2019‐00659) and was conducted in accordance with the principles of the Declaration of Helsinki.

## Consent

Written informed consent was obtained from all participants or from their legal guardians prior to participation in the study.

## Conflicts of Interest

The authors declare no conflicts of interest.

## Data Availability

The data that support the findings of this study are available on request from the corresponding author. The data are not publicly available due to privacy or ethical restrictions.
